# Lesion Preparation Complete – You May Now Deploy Your Stent

**DOI:** 10.1016/j.jscai.2025.103604

**Published:** 2025-05-01

**Authors:** Eric S. Rothstein, Nathan L. Crain, Jarrod D. Frizzell

**Affiliations:** a Heart and Vascular Center at Dartmouth Hitchcock Medical Center, Lebanon, New Hampshire; b Heart and Vascular Center at The Christ Hospital, Cincinnati, Ohio

**Keywords:** coronary calcium, intravascular imaging, intravascular lithotripsy, intravascular ultrasound

“Build a time machine, and stop yourself from putting in the stent!” was the advice I received when asking a senior colleague about my options for optimizing a freshly implanted but significantly underexpanded stent following postdeployment intravascular lithotripsy (IVL) and high-pressure balloon inflations. Multiple trials have demonstrated superior outcomes with intravascular imaging (IVI)-guided percutaneous coronary intervention (PCI) when compared with angiographic guidance.^1^, ^2^, ^3^, ^4^ Although stent sizing and stent edge characteristics (dissection and plaque burden) are important when using IVI, the biggest determinate of outcomes is final minimal stent area.^1^, ^2^, ^3^, ^4^ Accordingly, there has been an increased focus surrounding lesion preparation to achieve adequate stent expansion (ASE).

Scoring systems exist for both intravascular ultrasound (IVUS)^5^ and optical coherence tomography^6^ for quantitative analysis of calcium burden, and recommendations exist surrounding using these scores to guide calcium modification for vessel preparation.^7^ However, a fundamental concern is the lack of a reliable IVI-based metric confirming adequate lesion preparation. Currently, the options are limited to either the relatively insensitive marker of calcium fractures or the classic technique of fluoroscopic judgment of balloon expansion.

We seem to understand well which lesions ought to be modified, but not *how well* our modification techniques have actually worked. A contemporary marker for adequate lesion preparation is necessary to achieve the exceptional outcomes that our field strives to deliver. As my colleague implied, you cannot undo stent deployment. In this setting, Oliveri et al^8^ propose evaluating coronary artery compliance (CACom) and the subsequent change in coronary artery compliance (ΔCACom) following lesion preparation as novel metrics evaluating adequate calcium preparation.

In this issue of JSCAI, Oliveri et al describe their experience evaluating PCI results from a subset of 49 patients (of 455 total patients) in the prospective, multicenter BENELUX-IVL registry. These patients all underwent IVUS-guided PCI with imaging performed both before and after the use of IVL, as well as after stent deployment, to examine the relationship between specific predictors of adequate lesion preparation and ASE.

Patients had evidence of severe target lesion calcification (all had calcium arcs >305°). The team evaluated CACom and ΔCACom as predictors of lesion preparation leading to ASE. CACom was calculated by identifying the site of minimal luminal area, measuring the difference in area at that segment between systole and diastole, and dividing that value by the difference in aortic pulse pressure. ΔCACom is the difference in CACom before and after lesion preparation with IVL. These markers were evaluated with regard to their correlation with achieving ASE compared with various other patient comorbidities, lesion characteristics, and procedural steps performed in both univariate and multivariable regression models. Multiple markers for ASE were utilized, including minimal stent area (MSA) >5.5 mm^2^ and/or MSA >80% of the distal reference vessel. While the absolute CACom measured post-IVL did not show a significant correlation with ASE, the ΔCACom seemed to be the best predictor of stent expansion of all variables tested, although the correlation with an MSA >5.5 mm^2^ was modest (*r* = 0.42).

Performing a high-quality, registry-based imaging study is challenging, and several challenges limited this study. All measurements were made using a manual pullback 3.5F IVUS catheter. This could lead to significant selection bias, as calcified vessels are notoriously challenging to deliver equipment through, and patients were not included in the study if IVUS was not performed prior to IVL. Additionally, manual pullback with this catheter could cause the device to skip back through heavily calcified lesions. Combined with the absence of angiographic coregistration of the IVUS images, there are concerns about measuring the exact same vessel segment in subsequent images. In addition, possibly related to selection bias, the disease complexity in this patient population was in the low-to-intermediate range (95% of patients had a SYNTAX score ≤26), and vessels treated were all relatively large (all vessels had a diameter ≥3.5 mm). Unsurprisingly, an MSA >5.5 mm^2^ was achieved in all but one of the 49 patients studied, making it challenging to evaluate the predictive capabilities of CACom or ΔCACom for stent expansion. However, when ASE was defined by achieving an MSA >80% of the distal reference vessel, which only occurred in 45% of patients, ΔCACom was a highly significant predictor of stent expansion. Despite these limitations, the results of this study remain useful and intriguing, as it not only evaluated novel markers for vessel preparation but demonstrated the effect of IVL on CACom.

Despite the clear benefits of IVI-guided PCI, utilization in the USA remains <20%.^9^ The Level 1A recommendation to use IVI in the 2025 ACC/AHA/ACEP/NAEMSP/SCAI acute coronary syndrome guidelines^10^ should provide further encouragement to drive more operators to use IVI; however, a major barrier to uptake may be uncertainty as to how to best use the wealth of information provided by IVI, especially with regard to calcium. Understanding the compliance of the vessel being treated is fundamental for safe and effective lesion preparation. The development of imaging-derived measurements surrounding the effects of various calcium modification techniques on lesion compliance is vital to advancing PCI of calcified vessels. While it is exciting to see new potential markers such as CACom and ΔCACom emerging, utilizing them in “real-time” in contemporary practice as suggested by the authors remains challenging. The amount of work necessary to make multiple accurate measurements throughout the entire calcified lesion in both systole and diastole currently represents a significant barrier to evaluating CACom. Fortunately, this is yet another space in the field of interventional cardiology in which artificial intelligence (AI) may augment our understanding of vessel anatomy. This could be complementary to AI used in IVI^11^ that is already commercially available (eg, AVVIGO, Boston Scientific). Should further studies verify the importance of CACom as a marker of adequate lesion preparation, one could easily imagine integrating such AI-derived CACom evaluation of the entire lesion into an algorithmic assessment of prestent IVI. We eagerly await further research evaluating the limitations of this study and addressing the utility of CACom modification prior to mainstream adoption.
